# Draft Genome Sequence of Bifidobacterium longum BB-79, a Strain That Produces Acid Exopolysaccharide

**DOI:** 10.1128/MRA.01238-18

**Published:** 2018-11-15

**Authors:** Linyan Cao, Junlin Yu, Huahai Chen, Yeshi Yin

**Affiliations:** a College of Chemistry and Bioengineering, Hunan University of Science and Engineering, Yongzhou, China; Queens College

## Abstract

The surface exopolysaccharide of Bifidobacterium spp. was found to be involved in many processes, including bacterial colonization and host immune modulation. Here, we report the draft genome sequence of an exopolysaccharide-producing strain of Bifidobacterium longum, BB-79.

## Abstract (precis)

The surface exopolysaccharide of Bifidobacterium spp. was found to be involved in many processes, including bacterial colonization and host immune modulation.

## ANNOUNCEMENT

The Bifidobacterium genus contains well-recognized probiotic organisms besides Lactobacillus spp. (1). Cell surface exopolysaccharides (EPSs) of Bifidobacterium spp. were reported to play many important roles, such as protection against gastrointestinal pressure (2) and modulation of the host immune response (3, 4). However, the gene clusters and structures of EPSs in bifidobacteria were found to be different among strains (5).

Bifidobacterium longum strain BB-79 (ATCC 51870, CICC6187) was isolated from human feces in Japan (6). We obtained this strain from the China Center of Industrial Culture Collection (CICC) and cultured it in peptone yeast glucose (PYG) broth. The genomic DNA was extracted with the Omega E.Z.N.A. bacterial DNA kit and sent to Shanghai Majorbio Bio-Pharm Biotechnology, Co., Ltd., (Shanghai, China) for sequencing. The genomic DNA was quality checked by electrophoresis before being sheared into 400 to 500-bp fragments by a Covaris M220 instrument. Then, a paired-end library (2 × 300 bp) was constructed with a TruSeq DNA sample prep kit. The genome sequencing was performed on an Illumina MiSeq platform with MiSeq reagent kits v3 or v2. The reads were quality checked by an analysis of the G+C deviation and base error rate. Low-quality reads (read value, <Q20; N content, >10%; or read length, <25 bp) were cut. The sequence reads were assembled using SOAPdenovo v2.04 with different parameters, and the best results were adopted (7). The GapCloser v1.12 module with default settings was applied to fill the gap and for base correction. Gene prediction was done using Glimmer 3.02 as instructed (8). Annotation was done by comparing BLAST (2.2.28+) against the nonredundant (nr), search tool for the retrieval of interacting genes (String), and gene ontology (GO) databases.

A total of 10,042,427 × 2 raw reads and 3,032,812,954 bases were obtained from the MiSeq instrument, with the Q20 of sequencing being 92.67%. Generally, the draft genome of Bifidobacterium longum BB-79 contains 23 scaffolds (24 contigs), with a G+C content of 59.87%. The total length of all contigs is 2,206,088 bp; the largest scaffold is 487,798 bp; and the *N*_50_ and *N*_90_ values of the scaffolds are 347,030 bp and 80,480 bp, respectively. In total, 1,923 genes, 59 tRNAs, and 1 complete 5S-23S-16S rRNA gene cluster were found. The G+C content in the gene region is 60.73%, higher than that in the intergenic region (54.03%).

### Data availability.

This draft whole-genome shotgun project has been deposited at DDBJ/ENA/GenBank under the ENA run accession number ERR2843385. The BioProject accession number is PRJEB28066.
